# Use of Monoterpenes as Potential Therapeutics in Diabetes Mellitus: A Prospective Review

**DOI:** 10.1155/2023/1512974

**Published:** 2023-11-15

**Authors:** Leonardo da Rocha Sousa, Nildomar Ribeiro Viana, Angélica Gomes Coêlho, Celma de Oliveira Barbosa, Débora Santos Lula Barros, Maria do Carmo de Carvalho e Martins, Ricardo Martins Ramos, Daniel Dias Rufino Arcanjo

**Affiliations:** ^1^LAFMOL–Laboratory of Functional and Molecular Studies in Physiopharmacology, Department of Biophysics and Physiology, Federal University of Piaui, Teresina, Brazil; ^2^LaBME–Laboratory of Molecular Biology and Epidemiology, Federal Institute of Education, Science and Technology of Piauí–Campus Teresina Central, Teresina, Brazil; ^3^Department of Pharmacy, University of Brasilia, Brasília, Brazil; ^4^LaPeSI–Information Systems Research Laboratory, Department of Information, Environment, Health and Food Production, Federal Institute of Piaui, Teresina, Brazil

## Abstract

Monoterpenes are secondary metabolites of plants belonging to the terpenoid class of natural products. They are the most abundant components of essential oils that are generally considered to have various pharmacological properties. These compounds are reported to have antidiabetic effects in recent years. Due to nature's complex biosynthetic machinery, they also exhibit a reasonable degree of structural complexity/diversity for further analysis in structure-activity studies. Therefore, monoterpenes as antidiabetic agents have been investigated by recent in vitro and in vivo studies extensively reported in the scientific literature and claimed by patent documents. The purpose of this survey is to provide a comprehensive and prospective review concerning the potential applications of monoterpenes in the treatment of diabetes. The data for this research were collected through the specialized databases PubMed, Scopus, Web of Science, and ScienceDirect between the years 2014 and 2022, as well as the patent databases EPO, WIPO, and USPTO. The research used 76 articles published in the leading journals in the field. The main effect observed was the antidiabetic activity of monoterpenes. This review showed that monoterpenes can be considered promising agents for prevention and/or treatment of diabetes as well as have a marked pharmaceutical potential for the development of bioproducts for therapeutics applications.

## 1. Introduction

Diabetes mellitus is a chronic disease that affects about 3% of the world's population and its prevalence has increased given the aging population. In 2015, the International Diabetes Federation (IDF) estimated that one in 11 adults aged 20 to 79 years had type 2 diabetes mellitus. Diabetes mellitus ranks ninth among diseases that cause loss of healthy life years. Around 422 million people worldwide have DM and 1.6 million deaths are directly attributed to the disease each year [1]. There has also been an increase in the number of deaths from DM between 1990 and 2019, from 1,278,866 to 2,988,924 (an increase of 133.71%). For disability-adjusted life years (DALYs), growth was observed from 28,586,671 in 1990 to 70,888,154 in 2019 (148% increase). In Brazil, the scenario is similar, with DM accounting for 43,787 deaths in 1990 and 107,760 in 2019 (7.64% of the total), as well as causing 1,730,460 DALYs in 1990 and 3,750,735 in 2019 (5.73% of the total) [2].

Diabetes mellitus (DM) has a complex and multifactorial etiology, involving genetic and environmental components, resulting from altered insulin production by the pancreas and/or inability to adequately exert its function in the body [3]. Diabetes can be classified into four main types: (a) type 1 diabetes (T1D), characterized by the autoimmune destruction of pancreatic *β* cells, causing deficiency in insulin production; (b) MODY type diabetes, caused by idiopathic insulin deficiency; (c) type 2 diabetes (T2D), caused by the progressive loss of insulin secretion combined with insulin resistance; and (d) gestational diabetes characterized by hyperglycemia of varying degrees, diagnosed during pregnancy [4].

Type 1 diabetes (T1D) presents itself as an autoimmune disorder marked by the destruction of *β* cells in the islets of Langerhans of the pancreas, mediated by T lymphocytes. This destruction leads to an almost complete loss of the ability to synthesize insulin [5]. As a result of this depletion of *β* cells, the body loses the ability to regulate blood sugar levels, requiring, as a classic alternative treatment, the administration of exogenous insulin. Besides, T2DM is characterized by the progressive and irreversible loss of *β* cell function [6].

Conventional treatment of DM1 continues to be based mainly on the therapeutics use of insulin, more recently making use of various synthetic analogues of the hormone with different pharmacokinetic profiles. In addition to insulin and its analogues, some noninsulin drugs, such as biguanides, are used as therapeutics adjuvants in the treatment of DM1. Currently, metformin is the most prescribed noninsulin drug in Brazil, although the percentage of patients who use this therapy does not reach 5% of the total [7]. Current treatment of T2DM basically consists of changes in diet and lifestyle with the progressive addition of more complex antidiabetic regimens (including metformin, sulphonylureas, sodium-glucose cotransporter-2 inhibitors, thiazolidinediones, and so on) and eventually supplemental insulin therapy [8].

DM evolves with micro- and macrovascular complications, which result in repercussions on target organs, such as the heart, blood vessels, eyes, kidneys, and brain [9]. One of the reasons to these outcomes is correlated to a low total adherence (1.4%) to the suggested clinical treatment, as pointed out by Faria et al. [10] who found an adherence of 84.4% of diabetics to drug treatment, 58.6% to regular physical activity practice, and 3.1% to a diet plan appropriate to the disease.

The low adherence to DM treatment has a huge economic impact on today's society, affecting not only health systems but also individuals and their families, in addition to generating a loss of quality of life for the patient, with a high degree of limitation in work and leisure activities [11]. In this context, patients with DM have a higher risk of presenting polypharmacy (35.8%) than other patients that can lead to risk of drug interactions (15.1%) and impairment to the diabetic's glycemic control [12]. The challenge is to reduce polypharmacy, especially in the elderly, choosing safe, effective options without risk of interactions. In this sense, the use of food supplements (vitamins, phenolic compounds, omega 3, and other bioactive compounds) has gained space, especially in the treatment of T2D. In the search for an antioxidant and anti-inflammatory effect adjacent to the hypoglycemic effect, the search for bioactive compounds has gained increasing importance, among which we highlight the monoterpenes.

Several studies have already proven the therapeutics importance of products of natural origin in the control, treatment, and prevention of several diseases. Substances called phytochemicals are extracted from these natural products. Among the phytochemicals most studied for therapeutics purposes, polyphenols, flavonoids, alkaloids, tannins, coumarins, and terpenoids stand out, the latter being the object of study in this work. Among the main pharmacological activities attributed to many of these substances, especially to terpenes, anti-inflammatory, gastrointestinal tract protective, cardioprotective, antioxidant, and antidiabetic activity stand out [13]. Terpenoids are secondary metabolites of various types of plants, synthesized by the mevalonic acid pathway and contribute to their direct defense mechanism and their flavor profile [14].

Terpenes are the most abundant natural products, present in higher plants, citrus, conifers, and eucalyptus, and are widely distributed in the leaves, flowers, stems, and roots of these plants. Terpenes show significant structural variation, including linear hydrocarbons or carbocyclic skeletons. Terpenes undergo oxygenation, hydrogenation, or dehydrogenation to form terpenoids [15]. The classification of terpenes is based on the isoprene units proposed by Wallach in 1887 (C5H8), a 5-carbon compound that forms the skeletons of terpenes, and can be didactically described as follows: hemiterpenes are the simplest, with a single unit of isoprene; monoterpenes are highly diverse and have two isoprenoid units; sesquiterpenes are abundant natural compounds with three isoprene molecules; diterpenes are nonvolatile C20 hydrocarbons derived from four isoprene units; triterpenes are derived from the C30 precursor, squalene, made up of two molecules of sesquiterpenes; and tetraterpenes (carotenoids) are 8-isoprene units [16].

Monoterpenes occur in monocotyledonous and dicotyledonous angiosperms, fungi, bacteria, and gymnosperms and have two isoprenoid units. They are aromatic compounds responsible for the odor of many flowers and fruits. Monoterpenes include acyclic, monocyclic, and bicyclic forms. They are widely used as active ingredients for agricultural, pharmaceutical, cosmetic, and food applications. Pinenes, carveol, camphor, menthol, and limonene, for example, are active ingredients in a wide variety of industrial applications.

**Figure 1:**
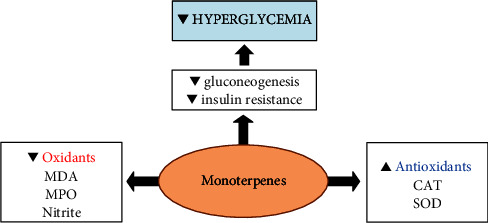
Summary of effects of monoterpenes on diabetes.

Monoterpenes, like sesquiterpenes and diterpenes, are secondary metabolites because they are classified as nonessential for viability and mediate important interactions between plants and their environment [17]. Several monoterpenes are widely used in the agricultural, cosmetic, and food industries and as a general antiseptic in medical practice [18], in addition to clinical use for their pharmacological properties, including antifungal, antibacterial, antioxidant, anticancer, and antispasmodic [19–23]. Monoterpenes can also have significant effects in diabetes by promoting hypoglycemic activity [24]. Studies have shown that monoterpenes include a wide variety of substances that possess antioxidant activity [25], which may prevent diabetic complications associated with oxidative stress [26, 27]. The general effects of monoterpenes on diabetes are schematized in Figure 1.

In this context, monoterpenes may be useful as agents for the prevention and/or treatment of diseases such as diabetes, a pathology with high mortality rates. In an attempt to reduce this impact, the search for substances of natural origin that have an antidiabetic effect has intensified. In this field, the study of the effects of some monoterpenes on diabetes stands out, such as limonene and carveol [28]. Considering the functional multicomplexity linked to diabetes, monoterpenes draw attention for demonstrating in pharmacological studies diverse and interesting in vitro and in vivo effects of several of their chemical constituents present in essential oils of medicinal plants on diabetes. Thus, the objective of this work was to carry out a bibliographic review of the studies that deal with the effects of monoterpenes on diabetes, highlighting the types of research carried out (in vitro and in vivo), experimental models used, and main results obtained. In addition, we carry out technological prospecting for product patents containing monoterpenes developed for the treatment and/or prevention of diabetes and its main complications.

The technological prospection of patents and the inclusion in the research of more recent articles on the subject: monoterpenes as a therapeutics alternative to conventional treatments for diabetes, not yet mentioned in previous reviews, undoubtedly constitute factors of scientific innovation of this work.

## 2. Methodology

This systematic review used a literature search to find articles published in the last eight years. The search was conducted using the specialized databases PubMed, Scopus, Web of Science, and ScienceDirect, using a combination of the English descriptors: “Monoterpenes” and “Diabetes.” A total of 1410 articles were found, of which 76 articles were used for the literature survey. The following inclusion criteria were adopted: years 2014 to 2022, clinical and nonclinical studies using monoterpenes in the prevention and/or treatment of diabetes, written in English, full text in electronic media, and articles that presented results of monoterpenes alone or in combination. Articles that were found in more than one database (duplicates) were included in the final count of articles used in the study only once. As for the exclusion criteria, we did not use abstracts, monographs, theses, thesis chapters, books, book chapters, congress or conference proceedings, and technical and scientific reports, in addition to excluding articles considered “irrelevant” to the study after a thorough reading.

The search for patents was conducted in the European Patent Office (EPO), World Intellectual Property Organization (WIPO), and United States Patent and Trademark Office (USPTO) databases using the combination of descriptors in English “monoterpenes” and “diabetes,” in addition to a search in the Brazilian National Institute of Industrial Property (INPI) database with descriptors in Portuguese. No time frame was established for the search.

**Figure 2:**
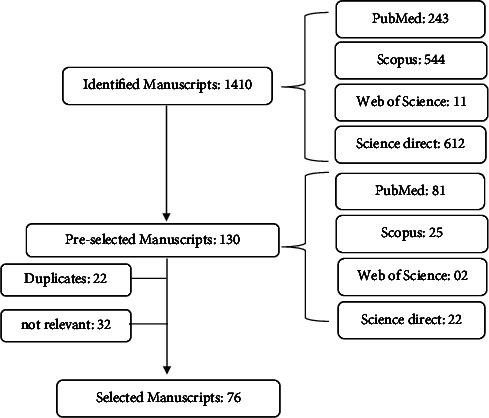
Flowchart of manuscript selection.

Figure 2 shows the flowchart of the article selection process.

## 3. Important Aspects of the Analyzed Manuscripts

### 3.1. Characterization of the Manuscripts

The data from the 76 manuscripts used in this review were entered into the statistical program SPSS (Statistical Package for the Social Sciences) version 24 where a statistical analysis was performed in order to explore the main findings in common among the various studies used. The following variables were analyzed in the SPSS database: (1) author of the study; (2) year of publication of the study; (3) journal in which the article was published; (4) type of monoterpene studied; (5) type of study; (6) the study model; (7) the study objective; (8) the dose used; (9) the concentration; (10) the route of administration; (11) the activity/effect of the monoterpene studied; (12) the mechanism(s) of action; (13) the markers used in the study; and (14) the conclusion of the study. Manuscripts from 58 different authors were analyzed, where 8 different authors presented 2 articles each. The research was conducted between the years 2014 and 2022. The most prevalent years were 2017 with 17 articles (22.37%) and 2018 with 16 articles (21.05%) (Table 1).

The manuscripts selected were published in 54 different journals, where the journal with the highest prevalence was Biomedicine & Pharmacotherapy with 8 manuscripts (10.53%). Twenty different monoterpenes were analyzed: geraniol, limonene, catalpol, cymene, geniposide, gentiopicroside, paeoniflorin, borneol, carvacrol, carvone, menthol, myrtenal, thymol, genipin, pinene, citronellol, citral, eucalyptol, swertiamarin, and loganin. The most studied monoterpenes were paeoniflorin with 17 articles (22.07%) and catalpol with 07 articles (9.09%) (Table 2).

As for the type of study, in vivo trials were the most prevalent being recorded in 54 manuscripts (71.05%), while in vitro studies appeared in 22 studies (28.95%), with 4 articles from in vivo trials that also did in vitro trials. A synthesis of the studies was performed according to their characteristics. The main study models were rats with 33 manuscripts (43.42%), mouse with 21 studies (27.63%), and cells with 19 studies (25.00%).

In in vitro studies, the concentrations used of the monoterpenes analyzed ranged from 0.01 to 200 *µ*M, with 57.70% of the manuscripts evaluating monoterpenes at doses lower than 50 *µ*M. The in vivo manuscripts presented doses ranging from 5 to 500 mg/kg, with 49.10% of studies with doses less than or equal to 50 mg/kg and 47.27% of studies testing doses ranging from 50 to 200 mg/kg. It is worth noting that in this universe of studies, there was a greater emphasis on testing the doses of monoterpenes at 50 mg/kg and 100 mg/kg. The main activity of the monoterpenes analyzed was antidiabetic, found in 45 manuscripts (59.21%). The main route of administration used in the analyzed articles was oral with 36 studies (67.92%).

### 3.2. Pharmacological Activity of Monoterpenes, Observed Effects, and Underlying Markers

The main pharmacological activities found in the studies reviewed were antidiabetic, antihyperlipidemic, antioxidant, anti-inflammatory, hepatoprotective, nephroprotective, cytoprotective, cardioprotective, vasorelaxant, and hypoglycemic. The main monoterpenes identified in this review as well as the type of study, study model, the observed effects, and the markers involved are listed in Tables 3 and 4.

Paeoniflorin (PF) ameliorated the inhibitory effect caused by streptozotocin (STZ) on cell viability and insulin secretion capacity in INS-1 cells, reduced caspase-3 activity and Bax expression, and induced Bcl-2 expression in STZ-treated INS-1 cells. Treatment with paeoniflorin resulted in a decrease in the production of reactive oxygen species and malondialdehyde (MDA) and an increase in superoxide dismutase (SOD) activity in STZ-treated INS-1 cells. In addition, this monoterpene inhibited the phosphorylation of p38 and JNK, which is induced by STZ in INS-1 cells, and suppressed the activation of p38 MAPK and JNK pathways in STZ-treated INS-1 cells. Paeoniflorin may be a natural antidiabetic agent by ameliorating pancreatic *β* cell injury through inhibition of p38 MAPK and JNK signaling pathways [42]. For Wang et al. [96], paeoniflorin significantly attenuated STZ-induced mitochondrial dysfunction and improved impaired insulin signaling by positively regulating p-PI3K and pAkt protein expression, while negatively regulating p-IRS-1 protein expression.

Paeoniflorin significantly decreased serum insulin and glucagon levels, improved insulin sensitivity and serum lipid profile, and alleviated hepatic steatosis in fructose-fed rats. In addition, it increased the phosphorylation level of AMP-activated protein kinase (AMPK) and protein kinase B (PKB/AKT) and inhibited the phosphorylation of acetyl-coenzyme A carboxylase 1 in the liver. Paeoniflorin also increased the mRNA of hepatic carnitine palmitoyltransferase I (CPT1) and protein expression and decreased the mRNA expression of regulatory element binding protein (SREBP) 1c, stearyl-coenzyme A decarboxylase (SCD)-1, and fatty acid synthase (FAS). In addition, it significantly increased the expression of the hepatic tumor suppressor protein serine/threonine kinase 1 [95]. According to Chen et al. [47], paeoniflorin can decrease the expression of LC3II/LC3I and reduce the number of autophagosomes. In addition, PF inhibited autophagy, at least in part, through inhibition of RAGE and positive regulation of p-mTOR level against AGEs-induced mesangial cell dysfunction. Paeoniflorin can decrease the urinary albumin excretion rate and inhibit macrophage infiltration and activation through blocking the TLR2/4 signaling pathway. The said monoterpene reduced AGEs-induced TLR2/4 activation and inflammatory responses, indicating that it prevents macrophage activation by inhibiting TLR2/4 signaling expression in type 2 diabetic nephropathy [94].

Paeoniflorin increases MMP and ATP levels as well as attenuates NF-*κ*B p65 expression mainly due to its antioxidant capacity, suppressing ROS production. In addition, it can suppress HIF-1*α* and VEGF protein expression through a decrease in ROS production via negative regulation of Nox2/Nox4 expression. It is worth noting that paeoniflorin protects against oxidative damage mainly through a mechanism involving a decrease in ROS production by inhibiting Nox2/Nox4 and RAGE expression in addition to restoring ATP depletion and mitochondria dysfunction via suppression of ROS and downregulating HIF-1*α*/VEGF, possibly via the ROS-NF-*κ*B axis [46]. As per Sun et al. [92], paeoniflorin treatment inhibited hyperphosphorylation of tau protein in the hippocampus. This function was correlated with its ability to reduce brain inflammatory cytokines (IL-1*β* and TNF-*α*) by decreasing suppressive cytokine signaling 2 (SOCS2) expressions and promoting insulin receptor substrate-1 (IRS-1) activity. In addition, it significantly promoted phosphorylation of protein kinase B (Akt) and glycogen synthase kinase-3*β* (GSK-3*β*) and had beneficial effects on alleviating diabetes-associated cognitive deficits through regulation of the SOCS2/IRS-1 pathway.

Paeoniflorin also has an action at the level of increasing the expression of mitochondrial processing peptidase *α* (PMPCA) and small ubiquitin 1 (Sumo1) which are responsible for increasing mitochondrial Trx2 (thioredoxin 2) protein processing and increasing Trx2 levels, TrxR2, and Prx3 in the sciatic nerve of rats with diabetic neuropathy, a severe consequence of diabetes mellitus, thereby reducing demyelination as well as improving mechanical pain threshold, thermal pain threshold, motor nerve conduction velocity (MNCV), and sensory nerve conduction velocity (SNCV). Overall, these results suggest that paeoniflorin could provide protection for the diabetic neuropathy (ND) condition by regulating Trx2 [98]. Paeoniflorin is, therefore, a natural glycoside with antihyperglycemic effect.

Catalpol is a natural product isolated from *Rehmannia glutinosa* root, which has been reported to produce antidiabetic effect [30]. Catalpol possesses hypoglycemic effect via modulation of various gene expressions such as SOCS3, Irs1, Idh2, and G6pd2 [65]. Catalpol enhanced hepatic NADPH oxidase type 4- (Nox4-) mediated oxidative stress and activated hepatic AMP-activated protein kinase (AMPK) and phosphatidylinositol 3-kinase (PI3K) via AKT in vivo and in vitro. The suppressive effect of catalpol on NOX4 was weakened by silencing AMPK with siRNA. Catalpol improved hepatic insulin resistance in type 2 diabetes through action on the AMPK/NOX4/PI3K/AKT pathway [30]. Catalpol has a protective effect on the endothelium in type 2 diabetes mellitus and its mechanism may be associated with the negative regulation of Nox4 and p22phox expression, inhibiting the oxidative stress reaction response [67]. According to Xu et al. [29], catalpol treatment in mice reduced blood glucose and improved insulin sensitivity via phosphatidylinositol-3-kinase (PI3K)/via protein kinase B (AKT) activation. Furthermore, catalpol-treated mice exhibited increased myogenesis, as evidenced by increased expression of myogenic differentiation (MyoD), myogenin (MyoG), and myosin heavy chain (MHC). In vitro experimental results showed that catalpol increased glucose uptake via activation of PI3K/AKT pathway that was dependent on MyoD/MyoG-mediated myogenesis.

Oral administration of catalpol at 100 mg/kg significantly improved fasting glucose and insulin levels, glucose tolerance, and insulin tolerance. In addition, macrophage infiltration of adipose tissue was markedly reduced by catalpol. Interestingly, catalpol also significantly reduced the mRNA expressions of proinflammatory M1 cytokines but increased the expressions of anti-inflammatory M2 genes in adipose tissue. Simultaneously, catalpol significantly suppressed c-Jun NH2-terminal kinase (JNK) and nuclear factor-kappa B (NF-*κ*B) signaling pathways in adipose tissue. Catalpol can improve HFD-induced insulin resistance in mice by attenuating adipose tissue inflammation and suppressing JNK and NF-*κ*B pathways [66]. In the manuscript of Yang et al. [48], catalpol treatment abrogated the elevated expression of Grb10 in diabetic kidneys. IGF-1 mRNA levels and IGF-1R phosphorylation were significantly higher in the kidneys of treated diabetic mice than those in untreated diabetic mice. Elevated Grb10 expression may play an important role in nephropathy pathogenesis through suppression of the IGF-1/IGF-1R signaling pathway, which may be a potential molecular target of catalpol for the treatment of diabetic nephropathy.

Geniposide is a naturally occurring iridoid glycoside. This monoterpene inhibited G6PC and PEPCK transcription in L02 cells and in mice and was able to inhibit FOXO1 transcriptional activity by inducing AKT phosphorylation at Ser473. Geniposide also alleviates high-fat diet-induced hyperglycemia in mice and can reduce blood glucose and suppress hepatic gluconeogenesis through regulation of the AKT-FOXO1 pathway [76]. Geniposide accelerates the degradation of Txnip (thioredoxin-interacting protein) by the proteasome pathway in pancreatic *β*-cells INS-1. Furthermore, the combination of geniposide and Txnip shows substantial synergistic effects to reduce glucose uptake, metabolism, and GSIS in INS-1 cells treated with high glucose [37]. The levels of the proinflammatory cytokines interleukin (IL)-1*β*, IL-6, and tumor necrosis factor *α* were decreased by geniposide in diabetic db/db mice. The expression levels of Rho, ROCK1, ROCK2, p-NF-*κ*Bp65, and p-I*κ*B*α* were significantly reversed by treatment with the said monoterpene, which demonstrated that geniposide exhibits a protective effect on diabetic liver inflammation [75]. For Hu et al. [74], Dusabimana et al. [77], and Chen et al. [79], geniposide acted through inhibition of ICAM-1, TNF-*α*, IL-1, and IL-6 expression and inhibition of NF-*κ*B activation by NF-*κ*B, IKK*α*, and I*κ*B*α* expression.

Geniposide protected *β*-cells through activation of Wnt signaling, increased expressions of TCF7L2 and O GLP-1R, activated AKT, inhibited GSK3 activity, and promoted catenin nuclear transactivation. The protective effect of geniposide was remarkably suppressed by siRNAs against *β*-catenin or by ICG001 (inhibitor of *β*-catenin/TCF-mediated transcription). Moreover, geniposide promoted *β* cell regeneration in vivo to normalize blood glucose on high-fat diet in mice. Increased *β* cells and proliferation were observed in the pancreas of diabetic mice treated with geniposide. More importantly, geniposide triggered formation of small cell clusters that was well correlated with increased TCF7L2 expression. In exocrine cells isolated from mouse pancreas, geniposide can induce differentiation of duct cells through positive regulation of TCF7L2 expression and activation of JAK2/STAT3 pathway [38].

In mice with diabetic retinopathy, geniposide inhibited the accumulation of reactive oxygen species, activating NF-*κ*B and Müller cells and being able to reduce inflammation via cytokine secretion, mediated by the Nrf2 pathway. Geniposide decreased hyperglycemia-induced damage to Müller cells and the blood-retinal barrier in the retinas of mice with diabetic retinopathy [78].

Carvacrol (CAR) is a monoterpene phenol that has good antioxidant activity. Increasing doses of CAR decreased tissue malondialdehyde (MDA) and 8-OH-dG levels as well as reduced lipase and amylase levels [59]. For Shoorei et al. [60], carvacrol treatment reduced the tissue activity of the enzymes superoxide dismutase (SOD) and glutathione peroxidase (GPx) and decreased elevated tissue malondialdehyde (MDA) levels. In addition, carvacrol significantly decreased Bax and increased Bcl-2 in gene and protein expression levels as well as reduced the rate of germ cell apoptosis.

Treatment with carvacrol in HFD-induced mice showed a decrease in plasma glucose and glycosylated hemoglobin as well as increased insulin and hemoglobin levels. The activities of carbohydrate metabolic enzymes such as glucose-6-phosphatase and fructose-1,6-bisphosphatase decreased, while the activities of glucokinase and glucose-6-phosphate dehydrogenase increased in the liver of HFD mice. The activities of liver marker enzymes such as aspartate aminotransferase, alanine aminotransferase, alkaline phosphatase, and gamma-glutamyl transpeptidase decreased in HFD mice [58]. For Ezhumalai et al. [57], carvacrol treatment further showed a decrease in total cholesterol (TC), triglycerides (TG), phospholipids (PL), and free fatty acids (FFAs) in plasma and tissues. In addition, decreased levels of very low-density lipoprotein cholesterol (VLDL-c) and low-density lipoprotein cholesterol (LDL-c) and increased levels of high-density lipoprotein cholesterol (HDL-c) were observed in the plasma of diabetic rats. Histopathological analysis of adipose tissues and immunohistochemical analysis of liver tissue showed that inflammatory cytokines (TNF-*α* and IL-6) were in agreement with biochemical parameters.

Carvacrol in STZ-induced rats decreased random plasma glucose and fasting plasma glucose levels significantly in a dose-dependent manner. Carvacrol at a dose of 20 mg/kg in diabetic mice caused the decrease in plasma triglyceride level and significantly reduced plasma LDH level, but not AST, ALT, or ALP, and there was increased activity of hexokinase (HK), 6-phosphofructokinase (PFK), and citrate synthase (CS) [61]. The study by Zhao et al. [62] showed improvement in glucose and insulin resistance of T2DM db/db mice treated with 10 mg/kg carvacrol. After treatment with carvacrol for 6 weeks, serum levels of total cholesterol, triglycerides, and LDL-cholesterol were markedly reduced, while level of HDL-cholesterol was significantly increased. Serum ALT and AST levels were significantly reduced in mice after the use of carvacrol, suggesting improvement of liver damage. Histological examinations confirmed that carvacrol can protect mouse liver by ameliorating T2DM-induced liver injury via mediating insulin, TLR 4/NF-*κ*B, and AKT1/mTOR signaling pathways.

Thymol is a monoterpene phenol with many pharmacological activities. Treatment with thymol decreased plasma glucose, insulin, insulin resistance, HbA1c, leptin, and adiponectin. Thymol supplementation significantly reduced plasma concentrations of triglycerides (TG), total cholesterol (TC), free fatty acids (FFAs), and low-density lipoprotein (LDL) and increased HDL-cholesterol [100]. Thymol inhibited the activation of transforming growth factor *β*1 (TGF-*β*1) and vascular endothelial growth factor (VEGF). In addition, it significantly increased antioxidants and suppressed lipid peroxidation markers in erythrocytes and kidney tissue. Thymol negatively regulates the expression level of sterol regulatory element-binding protein-1c (SREBP-1c) and reduces lipid accumulation in kidney. Histopathological study of kidney tissues showed that extracellular mesangial matrix expansion and glomerulosclerosis in diabetic mice were suppressed by thymol. Furthermore, thymol administration conferred remarkable protection against HFD-induced diabetic nephropathy [101].

Thymol also showed potent inhibitory activity against the enzyme aldose reductase as well as was able to significantly reduce oxidative stress, suggesting that thymol may be a potential therapeutics in preventing diabetic complications through its inhibitory and antioxidant activities [54]. Fang et al. [102] observed that thymol treatment significantly reversed body weight gain and peripheral insulin resistance. Thymol improved cognitive impairment and decreased HFD-induced A*β* deposition and hyperphosphorylation of tau protein in the hippocampus, which may correlate with inhibition of oxidative stress and inflammation in the hippocampus. Furthermore, thymol negatively regulates the level of P-Ser307 IRS-1 and thus increases the expression ofP-Ser473 AKT and P-Ser9 GSK-3*β*. The protective effects of thymol on cognitive deficits were associated with positive regulation of the nuclear respiratory factor (Nrf2)/heme oxygenase-1 (HO-1) pathway. Thymol exhibited beneficial effects on cognitive deficits by improving insulin resistance in the hippocampus and activating Nrf2/HO-1 signaling [102].

Thymol can markedly suppress inflammatory responses, apoptosis cells, and disordered cytoskeleton by restoring podocin expression. In western blot analysis, thymol indicated that it could restore the expression of RhoA, ROCK, vimentin, nephrin, and podocin and phosphorylation of p65 and I*κ*B*α*. In addition, si- RhoA also suppressed the expression of proinflammatory cytokines, ROCK, and vimentin and the phosphorylation of p65 and I*κ*B*α* [53].

In a 12-week treatment performed on streptozotocin- (STZ-) induced diabetic rats with induced diabetic neuropathy, thymol isolated from the fruit *Trachyspermum ammi* showed improvement in blood glucose, initially with a concentration of 308.27 ± 0.37 mg/dL and reducing on day 28 of use, respectively, to 129.01 ± 3.45 mg/dL on treatment with 10 mg/kg and to 117.12 ± 1.35 mg/dL with 10 mg/kg, in addition to improvement in lipid profile, such as triglycerides, LDL-cholesterol, and VLDL-cholesterol. The biomarker studies (SOD, NO, LPO, Na + K + ATPase, and TNF-*α*) further confirmed the protective action of thymol in diabetic neuropathy [103].

Genipin is the main active component of *Gardeniae fructu*s and has been shown to improve diabetes and insulin resistance in rat models. This monoterpene reduced body weight, food intake, and visceral fat mass. It improved dyslipidemia, glucose intolerance, insulin intolerance, adipocyte hypertrophy, and hepatic steatosis as well as reduced the serum level of tumor necrosis factor *α* in diet-induced obese mice. Moreover, genipin promoted lipolysis and *β*-oxidation of fatty acids by increasing gene expression of hormone-sensitive lipase and adipose triglyceride lipase in white adipose tissue (WAT) and peroxisome proliferator-activated receptor-*α* and carnitine palmitoyltransferase 1*α* in liver tissue. The said monoterpene also promoted browning of WAT by increasing mRNA and protein levels of uncoupling protein 1 and PRD1-BF1-RIZ1 gene. In addition, it inhibited the gene expressions of activin receptor kinase 7, tumor necrosis factor *α*, and interleukin 6 in WAT [71].

Genipin acts in regulating methylamine metabolism, energy metabolism, and amino acid metabolism [73]. Detailed analysis of altered metabolite levels indicated that genipin significantly ameliorated disruption in glucose metabolism, tricarboxylic acid cycle, lipid metabolism, and amino acid metabolism [72]. Genipin, a UCP2 inhibitor, dramatically increased oxidative stress, attenuated antioxidant capacity, and exacerbated cell apoptosis, accompanied by caspase-3 activation in rat proximal kidney cells (NRK-52E) incubated with high glucose. These data suggest that manipulation of UCP2 could be important in preventing oxidative damage in renal tubular epithelial cells induced by hyperglycemia in vitro [36].

Geraniol is an acyclic monoterpene alcohol found in medicinal plants and is traditionally used for various medical purposes, including diabetes. Administration of geraniol in a dose-dependent manner (100, 200, and 400 mg/kg b.c.) significantly improved insulin levels and Hb and decreased plasma glucose and HbA1C in diabetic rats. Geraniol at its effective dose (200 mg/kg b.w.) improved altered carbohydrate and metabolic enzyme activities. Treatment with geraniol in diabetic rats improved liver glycogen content, suggesting its potential hyperglycemic action. Geraniol supplementation was found to preserve the normal histological appearance of liver cells and pancreatic b-cells in diabetic rats [80].

Geraniol suppressed exaggerated oxidative stress, evidenced by prevention of 8-isoprostane increase. In diabetic heart tissue, geraniol prevented inhibition of catalase activity but did not affect heart SOD. Geraniol partially reduced hyperglycemia and prevented hypercholesterolemia but did not affect serum adiponectin level in diabetic animals. The findings suggest that geraniol provides a potent protective effect against diabetes-induced cardiac dysfunction. This beneficial effect can be attributed to the suppression of oxidative stress [81]. According to Prasad and Muralidhara [82], geraniol reduced oxidative markers, decreased levels of CP, Ca2+, and AChE, and restored the activities of enzymes, SDH, and CS.

Myrtenal is a monoterpene, a constituent of essential oils found mainly in herbs such as mint, pepper, and cumin. It exerts admirable pharmacological activities against many diseases, including diabetes. Myrtenal improves plasma glucose, pancreatic insulin, and lipid profiles (TC, TG, FFAs, phospholipids, LDL, VLDL, and atherogenic index) in addition to improving the histopathological feature of the liver [90]. Myrtenal treatment decreases plasma glucose levels, and increases insulin levels, as well as promote upregulation of GLUT2, Akt, and IRS2 in liver, and GLUT4, Akt, and IRS2 in skeletal muscle. Positive regulation of glucose transporters increases glucose uptake in liver and skeletal muscle [88].

Oral administration of myrtenal at doses of 20, 40, and 80 mg/kg body weight to diabetic rats resulted in a significant reduction in plasma glucose levels and glycosylated hemoglobin (HbA1c) and increased insulin and hemoglobin (Hb) levels. The altered activities of major metabolic enzymes involved in carbohydrate metabolism were noted, such as hexokinase, glucose-6-phosphatase, fructose-1,6-biphosphatase, and glucose-6-phosphate dehydrogenase, and the liver enzymes AST, ALT, and ALP of diabetic rats were significantly improved by myrtenal administration in STZ-induced diabetic rats. In addition, myrtenal treatment improved liver and muscle glycogen content in diabetic rats. Histopathological studies further revealed that the reduced islet cells were restored to near-normal conditions upon myrtenal treatment in STZ-induced diabetic rats. An alteration in liver architecture was also prevented by myrtenal treatment. The results suggest that myrtenal possesses antihyperglycemic and *β* cell protective effects and can be considered a potent phytochemical for development as a novel antidiabetic agent [89].

Cymene, a monoterpene commonly found in *Cuminum cyminum*, improved HbA1c and serum fructosamine, where levels were normalized. Nephropathic parameters such as albumin excretion rate, serum creatinine, and creatinine clearance rate were improved. Cymene treatment further improved the solubility profile of collagen. In in vitro studies, cymene inhibited the total fluorescence of AGEs and pentosidine. The glycation-specific decline in the *α*-helix content of BSA and increase in the B-sheet were prevented by cymene in vitro, implying its stabilizing effect. The results suggest that cymene could have therapeutics potential in preventing glycation-mediated diabetic complications [33]. Cymene strongly inhibits the formation of AGEs in a concentration-dependent manner, inhibiting protein glycation [32].

In a study with STZ-induced diabetic rats, p-cymene caused improvement in blood glucose and lipid profile (triglyceride, LDL-cholesterol, and VLDL-cholesterol), although the rates were close to the control group. p-Cymene administration improved the amount of malondialdehyde (MDA) in diabetic rats (*p* ≤ 0.001). Administration of p-cymene (100 mg/kg) can improve islet of Langerhans changes in diabetic rats and increase the expression of Akt, phospho-Akt, and mTOR, reducing liver and pancreatic injury [69].

Swertiamarin, a secoiridoid glycoside, is an antidiabetic drug with hypolipemic activity that improves insulin resistance in the condition of type 2 diabetes. Swertiamarin, at a concentration of 25 *μ*g/ml, decreased triglyceride content by 2-fold and effectively reduced LDH release activity (50%), protecting membrane integrity, preventing apoptosis evidenced by reduced Caspase 3 and PARP1 cleavage. Swertiamarin was observed to significantly increase the expressions of key insulin signaling proteins such as insulin receptor (IR), PI3K, and pAkt with concomitant reduction in p307 IRS-1. AMPK was activated by the action of swertiamarin, restoring insulin sensitivity in hepatocytes. Swertiamarin effectively modulated PPAR-*α*, an important potential regulator of carbohydrate metabolism, which in turn decreased levels of the gluconeogenesis enzyme PEPCK, further restricting hepatic glucose production and fatty acid synthesis. Cumulatively, swertiamarin targets potential metabolic regulators AMPK and PPAR-*α*, through which it regulates hepatic glycemic load, fat accumulation, insulin resistance, and ROS in hepatic steatosis, which emphasizes the clinical importance of swertiamarin in regulating metabolism and as a suitable candidate for the treatment of hepatic steatosis [51].

Swertiamarin at dosages of 15, 25, and 50 mg/kg bw for 28 days resulted in a significant reduction in fasting blood glucose, HbA1c, TC, TG, and LDL and increased levels of hemoglobin, plasma insulin, TP, body weight, and HDL. The effect of swertiamarin on carbohydrate metabolizing enzymes had normal therapeutic activity. Histopathological studies of the pancreas of diabetic rats treated with swertiamarin showed regeneration of islets when compared to STZ-induced diabetic rats. Swertiamarin has been shown to have antihyperglycemic, antihyperlipidemic, cytoprotective, and immunological reactivity. These properties allow for a wide range of treatment options for diabetes and its complications, making it a potentially effective oral antidiabetic [99].

Citral is a bioactive compound widely found in a variety of foods that are consumed daily. Citral treatment significantly decreased levels of triglyceride accumulation in a concentration-dependent manner. Cells treated with citral significantly suppressed the expression of PI3K/AKT, PPAR*γ*, SREBP-1c, FAS, CPD, TNF-*α*, IL-6, and MCP-1 in a dose-dependent manner [34]. According to Subramaniyan and colleagues [35], citral treatment (30 *μ*M) significantly decreased cellular cytotoxicity, ROS generation, DNA damage, and lipid peroxidation and increased antioxidant enzymes in high glucose-induced HepG2 cells. In addition, cells treated with high glucose showed increased expression of extracellular signal-regulated protein kinase 1 (ERK-1), N-terminal c-Jun kinase (JNK), and p38 in HepG2 cells. On the other hand, citral treatment significantly suppressed the expression of ERK-1, JNK, and p38 in high glucose-induced HepG2 cells. Citral protected against glucose-induced oxidative stress through inhibition of ROS-activated MAPK signaling pathway in HepG2 cells.

Loganin reduced renal to body weight ratio, 24 h urine protein levels, and serum urea nitrogen and creatinine levels in diabetic mice to different degrees. In addition, loganin improved the histology of the pancreas and kidney and alleviated structural changes in endothelial cells, mesangial cells, and podocytes in the renal cortex. In addition, loganin reduced AGE levels in serum and kidney and negatively regulated mRNA and protein expression of receptors for AGEs in the kidney in diabetic mice [84]. Loganin treatment decreased serum concentrations of IL-6 and TNF-*α*. Loganin treatment also significantly restored body weight gain and attenuated blood glucose changes in diabetic mice [85].

Loganin has been shown to have neuroprotective, antioxidant, and anti-inflammatory properties by reducing the intracellular generation of reactive oxygen species. At high blood glucose, there is increased expression of NLRP3, ASC, and caspase-1 protein; however, when treated with loganin, there was upregulation of these proteins that influences by decreasing NF-*κ*B phosphorylation and reducing oxidative stress, which may suggest a potential as a therapeutics in peripheral diabetic neuropathy [41] by improving insulin resistance, decreasing mRNA and protein levels of the proinflammatory factors IL-1 and TNF, and treating diabetes-associated complications [86].

Menthol significantly reduced blood glucose and glycosylated hemoglobin levels and significantly increased total hemoglobin, plasma insulin, and liver glycogen levels in diabetic rats. The altered activities of hepatic glucose metabolic enzymes and serum biomarkers of liver damage were restored to near normal. Pathological abnormalities in the hepatic and pancreatic islets of diabetic rats were significantly ameliorated by menthol. These effects were mediated by suppression of pancreatic *β* cell apoptosis and were associated with increased expression of antiapoptotic Bcl-2 and reduced proapoptotic Bax expression. Menthol alleviates STZ-NA-induced hyperglycemia via modulation of glucose-metabolizing enzymes, suppression of pancreatic cell apoptosis, and altered pancreatic and liver morphology. This uniqueness and paucity of any perceived adverse efficacy proposes the opportunity to use this monoterpene as an effective adjuvant in the treatment of diabetes mellitus [87].

Eucalyptol increased the podocyte expression of nephrin, podocin, FAT-1, CD2AP, and *α*-actinin-4 decreased by glucose. Oral administration of eucalyptol increased the induction of gap diaphragm proteins, *α*-actinin-4, and integrin *β*1 in diabetic kidneys and enhanced glomerular fibrosis and foot process deletion. Eucalyptol neutralized receptor induction of advanced glycation end products (RAGE) in podocytes with glucose or AGE-BSA and elevated the reduction of slit diaphragm proteins by AGE-BSA. Eucalyptol attenuated RAGE induction and AGE accumulation in diabetic kidneys. Blockade of ERK-c-Myc signaling increased the expression of negatively regulated nephrin and CD2AP in podocytes exposed to AGE. In this light, eucalyptol may be a potent agent that antagonizes diabetes-associated malformation [70].

Carvone reduced STZ-induced damage to liver and *β* cells of the pancreas. Carvone regulates carbohydrate metabolism by enhancing key enzymes in liver tissues of STZ-induced diabetic rats. The activities of carbohydrate metabolic enzymes, glycogen, and enzymatic antioxidants in pancreatic contents and liver markers were also altered. Daily oral administration of carvone (50 mg/kg b.w.) to diabetic rats for 30 days resulted in a significant decline in plasma glucose levels and HbA1c and significant improvement in hemoglobin and insulin levels. The reversed activities of carbohydrate metabolic enzymes, enzymatic antioxidants, and liver marker enzymes in diabetic rats were renewed to near-normal levels by carvone administration [63].

Alpha-pinene (AP) prevented ROS generation, lipid peroxidation, and DNA disruption, probably through its antioxidant property. AP also inhibited UVA-induced inflammatory mediators such as NF-*κ*B, TNF-*α*, and IL-6 expression in HaCaT cells. Furthermore, AP modulates NER proteins by activating p53 and p21 subsequently preventing the formation of UVA-induced cyclobutane pyrimidine dimers (CPDs). Alpha-pinene inhibits apoptotic cell death by preventing UVA-induced loss of mitochondrial membrane potential through modulation of Bax/Bcl-2 expression in HaCaT cells. Alpha-pinene prevents UVA-induced oxidative stress, inflammation, DNA damage, and apoptosis in human skin cells [50].

*β*-Pinene showed hypoglycemic and hypolipemic effects, which may involve some common mechanisms of glibenclamide. Oral administration of *β*-pinene in diabetic rats for seven days revealed an important anti-inflammatory effect that was the inhibition of inflammatory mediators that participate in the 2nd phase of the process, possibly decreasing the migration of leukocytes, indicating that its action may be linked to the inhibition of cytokine production [55].

Borneol is a monoterpene that acts by inhibiting Grb10 expression. In addition, treatment with this monoterpene is able to decrease blood glucose and aHbA1c, increase blood insulin, restore body weight loss, increase liver glycogen level, reverse the increase in levels of TC, TGs LDL-cholesterol, and VLDL-cholesterol induced by diabetes, restore the levels of urea, ALT, and AST, increase the antioxidant status of superoxide dismutase and catalase, and reduce glutathione in liver and kidney besides reducing the level of malondialdehyde [56].

Citronellol is a health beneficial monoterpene alcohol that occurs naturally in citrus oils. Citronellol improved insulin, hemoglobin, and liver glycogen levels, with significant decreases in glucose and HbA1C levels. Altered carbohydrate metabolic enzyme activities and liver and kidney markers were restored to near normal. Citronellol supplementation was found to be effective in preserving the normal histological appearance of liver cells and insulin-positive *β* cells in STZ mice. Citronellol administration attenuates hyperglycemia in STZ-induced diabetic rats by improving key carbohydrate metabolic enzymes and may be effective in combating diabetes mellitus [68].

Limonene (LM) is a monoterpene which is abundantly present in the peel of citrus fruits and can be obtained for a relatively low cost [104]. Several studies have been conducted to demonstrate the effects of this monoterpene. Hirota et al. [105] suggested that limonene may be effective in the treatment of bronchial asthma due to its anti-inflammatory effect, which probably occurs by inhibition of cytokines, production of reactive oxygen species (ROS), and inactivation of eosinophil migration. For Rozza et al. [106], limonene possesses gastroprotective activity in indomethacin- and ethanol-induced gastric ulcer models. In addition, limonene exhibits chemopreventive activity for breast, lung, skin, and liver cancer [107]. Limonene also possesses antinociceptive activity probably due to inhibition of synthesis or action of inflammatory mediators and antihyperglycemic activity in streptozotocin-induced diabetic rats [108, 109] in addition to vasorelaxant effect on isolated rat aorta [110].

Pretreatment of cells with limonene prevented methylglyoxal- (MG-) induced protein adduct formation, tumor necrosis factor-alpha and interleukin-6 release, mitochondrial superoxide production, and cardiolipin peroxidation. In addition, limonene increased glyoxalase I activity and glutathione and heme oxygenase-1 levels in the presence of MG. Pretreatment with limonene prior to MG exposure reduced MG-induced mitochondrial dysfunction by preventing mitochondrial membrane potential dissipation and adenosine triphosphate loss and reduced levels of adenosine monophosphate-activated protein kinase, peroxisome proliferator-activated receptor coactivator 1*α*, and nitric oxide and may prevent the development of diabetic osteopathy [40]. D-limonene treatment was found to significantly decrease DNA damage, GR enzyme activity, and MDA levels, significantly increase GSH levels and CAT, SOD, and GSH-Px enzyme activities, and alter lipid and liver parameters in diabetic rats [83].

Limonene treatment increased differentiation, lipid accumulation, and expression of adipogenic and lipogenic markers, such as C/EBP-*α*, C/EBP-*β*, PPAR*γ*, SREBP-1, RXR, FAS, and adiponectin. However, LM concentration at 10 *μ*M decreased adipogenesis and lipogenesis via regulation of key transcription factors. LM treatment increased Akt activation by increasing its phosphorylation, but p44/42 activation was not altered. MK-2206, a specific inhibitor of Akt, reduced the activation of Akt phosphorylation while LM treatment aborted the MK-2206-mediated inhibition of Akt activation. Limonene increased glucose uptake in differentiated adipocytes. Overall data suggested that LM treatment favored lipid storage and glucose uptake in adipocytes via activation of key transcription factors through activation of Akt phosphorylation in 3T3-L1 adipocytes [39].

### 3.3. Technological Prospecting

The database search returned a total of 22 patents to be analyzed. Among these, only five directly mentioned diabetes and/or its complications among the health problems that can be treated or prevented with the use of monoterpenes, which are detailed in Table 5. All the patents analyzed, according to the International Patent Classification, are intended for human needs (Section A), with medical (A61K) or food (A23) purposes.

Patents that only cited diabetes as a risk factor or disorder associated with other therapies that were subject to patent protection were excluded. In addition, the technological prospection excluded products with cosmetic purposes or those that reported agricultural applications related to this species.

Table 5 points out the lack of recent papers for the applicability of monoterpenes in the prevention or treatment of diabetes, since the identified patents date from 2012 or earlier years. This scenario is contradictory, since research released between the years 2014 and 2019 was included in the present review. It can therefore be assumed that the voluminous academic production in this theme has not yet been proportionally converted to patent protection object, so as to enable its industrial applicability and, consequently, commercial exploitation of the inventions by the pharmaceutical and food markets.

Among the patents analyzed, it is worth mentioning the work of Pei-Jung et al. [112], which claims ownership over the extract of *Toona sinensis* leaves, obtained by the supercritical fluid extraction method. The apolar fraction in question had its antidiabetic effect evaluated in vitro in adipocytic cell model and showed significant reduction of glucose concentration in the medium. In the composition of the extract, the presence of the monoterpenes *α*-pinene and *β*-pinene, as well as the derivative monoterpene, D(+)-limonene, stands out.

In the work of Shu Gang et al. [114], on the other hand, protection is claimed for different pharmaceutical forms containing the compound D-limonene, such as hard capsules, soft capsules, tablets, or powders. Extraction was performed by fractional distillation of the volatile oils of C*itrus limon* (L.) Burm. f., *Citrus reticulata* Blanco, and *Citrus aurantium*, among other plant species. This group evaluated the in vivo effect of D-limonene (2.4 ml/kg) and demonstrated its hypoglycemic action in rats up to 2 hours after oral administration, showing that preparations containing this monoterpene derivative are promising for diabetes control.

In the other patents evaluated, monoterpenes are cited as integrating pharmaceutical and/or dietary compositions, as well as for functional foods, useful for treating and generally preventing immune system [111] or metabolic disorders [113], as well as preventing aging processes and related conditions, such as atherosclerosis, hypertension, diabetes, tumors, and obesity. However, although the therapeutics potential of these inventions in the face of diabetes is clear, in these works, a clear relationship between the pharmacological actions played by monoterpenes in specific for diabetes mellitus is not established.

## 4. Conclusion

The hyperglycemia associated with diabetes mellitus and its associated diseases can be controlled with oral hypoglycemic agents, insulin, diet, and physical activity, but this review showed the therapeutics potential of monoterpenes with a wealth of specific effects and important markers that were able to act in blood glucose control and action to treat diabetic neuropathy, diabetic retinopathy, and possible liver damage associated with the evolution of this pathology. Geraniol, D-limonene, catalpol, cymene, geniposide, gentiopicroside, paeoniflorin, borneol, carvacrol, carvone, menthol, myrtenal, thymol, genipin, pinene, citronellol, citral, eucalyptol, swertiamarin, and loganin were the monoterpenes described with actions to reduce glycemia, triglycerides, total cholesterol, LDL-cholesterol, regeneration of islets of Langerhans, suppression of inflammatory cytokines, inhibition of reactive oxygen species, and reduction of oxidative markers, stimulating the search for new drugs. Therefore, the search for new drugs with antidiabetic and hypoglycemic actions that expand the phytotherapeutics collection is of utmost importance in order to unveil other activities of these monoterpenes, better elucidating their mechanisms of action and knowing their therapeutics doses.

## Figures and Tables

**Table 1:** Year of publication of the manuscripts and number of publications per year.

Year of publication	Frequency	%
2014	8	10.53
2015	8	10.53
2016	13	17.10
2017	17	22.37
2018	16	21.05
2019	2	2.63
2020	3	3.95
2021	4	5.26
2022	5	6.58
Total	76	100.0

**Table 2:** Monoterpenes used in the study.

Monoterpenes	Frequency	%
Borneol	1	1.30
Carvacrol	6	7.79
Carvone	1	1.30
Catalpol	7	9.09
Citral	2	2.60
Citronellol	1	1.30
Cymene	3	3.90
Eucalyptol	1	1.30
Genipin	4	5.19
Geniposide	8	10.38
Gentiopicroside	1	1.30
Geraniol	3	3.90
Limonene	3	3.90
Loganin	4	5.19
Menthol	1	1.30
Myrtenal	3	3.90
Paeoniflorin	17	22.07
Pinene	2	2.60
Swertiamarin	3	3.90
Thymol	6	7.79
Total	77	100.0

**Table 3:** In vitro antidiabetic effects of monoterpenes.

Monoterpenes	Model	Effects/markers	References
Catalpol	Myeloblasts C2C12 cells	Catalpol elevates MyoD/MyoG expression and improves skeletal muscle myogenesis. Enhanced myogenesis results in activation of the insulin signaling pathway in skeletal muscle cells (via PI3K/AKT), which may help improve glucose homeostasis.	[29]
Catalpol	Human hepatocellular carcinoma cells (HepG2)	Catalpol decreased in gluconeogenesis and increased in hepatic glycogen synthesis, through the regulation of the AMPK/NOX4/PI3K/AKT pathway	[30]
Catalpol	Human hepatocellular carcinoma cells (HepG2)	Catalpol attenuated the decrease in ATP levels and mitochondrial membrane potential, thereby reducing the formation of reactive oxygen species induced by elevated glucose levels	[31]
Cymene	Bovine serum albumin (BSA) glycation	Inhibition of BSA protein glycation	[32]
Cymene	Bovine serum albumin (BSA) glycation	Inhibition of BSA protein glycation	[33]
Citral	Mouse 3T3-L1 fibroblast preadipocytes	Inhibition of adipogenesis in 3T3-L1 adipocytes through modulation of adipogenic transcription factors and inflammatory markers	[34]
Citral	Human hepatocellular carcinoma cells (HepG2)	Protection against oxidative stress through inhibition of the ROS-activated MAPK signaling pathway in HepG2 cells	[35]
Genipin	Rat renal proximal tubular cells (NRK-52E)	A UCP2 (uncoupling protein-2) inhibitor, boosted oxidative stress, attenuated antioxidative capacity, and exacerbated cell apoptosis accompanied with caspase-3 activation in rat renal proximal tubular cells (NRK-52E)	[36]
Geniposide	Mouse INS-1 pancreatic *β* cells	Geniposide reduces apoptosis of pancreatic cells by inhibiting the Txnip protein	[37]
Geniposide	Pancreatic cells from C57BL/6J mice	Geniposide protected *β* cells against hyperglycemia and toxicity mediated by proinflammatory cytokines, through activation of *β*-catenin signaling and upregulation of TCF7L2 expression and activation of the JAK2/STAT3 pathway	[38]
Limonene	C2C12 skeletal muscle cells	Limonene induces osteoblast differentiation and glucose uptake through activation of p38MAPK and Akt signaling pathways	[39]
Limonene	Osteoblastic cells MC3T3-E1	Limonene reduced ROS, inflammatory cytokines, and mitochondrial dysfunction and increased AMPK, PGC-1*α*, and NO levels	[40]
Loganin	Schwann cell line RSC96 cells	Loganin attenuates hyperglycemia-induced Schwann cell pyroptosis by inhibiting ROS generation and NLRP3 inflammasome activation	[41]
Paeoniflorin	Mouse INS-1 pancreatic *β* cells	Paeoniflorin prevented oxidative stress and cellular apoptosis in STZ-treated INS-1 cells through inhibition of p38 MAPK and JNK pathways	[42]
Paeoniflorin	Human retinal pigmented epithelial cells (ARPE-19)	Paeoniflorin dose-dependently attenuated RAL-induced cell injury by reducing oxidative stress associated with Nox1/ROS, mitochondrial dysfunction, and endoplasmic reticulum (ER) stress in ARPE-19 cells	[43]
Paeoniflorin	Microglial cells BV2	Paeoniflorin suppressed expression of cytokine 3 signaling (SOCS3) and reduced MMP-9 activation, attenuating diabetic retinopathy in BV2 cells	[44]
Paeoniflorin	Cells	Suppression of ROCK activation and IRS-1 expression, promoting phosphorylation of Akt and GSK-3*β*	[45]
Paeoniflorin	Human umbilical vein endothelial cells (HUVEC)	Paeoniflorin reduced AOPP-induced oxidative damage in HUVECs, decreasing ROS production by inhibiting Nox2/Nox4 and RAGE expression, and restored ATP depletion and mitochondrial dysfunction via suppression of ROS	[46]
Paeoniflorin	Glomerular mesangial cells HBZY-1	Paeoniflorin attenuates mesangial cell damage induced by advanced glycation end products (AGEs) and combats autophagy through inhibition of RAGE and upregulation of p-mTOR level	[47]
Paeoniflorin	Schwann cell lineage (RSC96)	Paeoniflorin suppressed the oxidative stress of Schwann cells induced by hyperglycemia, decreasing ROS and MDA levels and increasing GST and GPX activity, promoted the dissociation of Nrf2 from Keap1, and upregulated the Nrf2 pathway	[48]
Paeoniflorin	Methylglyoxal- (MG-) induced MC3T3-EI osteoblastic cells	Paeoniflorin reduced MG-induced apoptosis and ROS formation in MC3T3-E1 osteoblastic cells. It increased GSH level and reduced MG-induced mitochondrial dysfunction.	[49]
Pinene	Human skin epidermal keratinocytes (HaCaT cells)	Pinene inhibited ROS formation, lipid peroxidation, and DNA breakdown through its antioxidant property. It also suppressed the expression of NF-*κ*B, TNF-*α*, and IL-6 in HaCaT cells	[50]
Swertiamarin	Human hepatocellular carcinoma cells (HepG2)	Swertiamarin increased the expression of key insulin signaling proteins such as IR, PI(3)K, and pAkt, with concomitant reduction in IRS-1, activated AMPK, modulated PPAR-*α*, and decreased levels of the gluconeogenic enzyme PEPCK	[51]
Swertiamarin	5-HT2 receptor	Normalize the mRNA expression of Glut 4, adiponectin, SREBP-1c, PPAR*γ*, LPL-1, and leptin. Increase in PI3K expression.	[52]
Thymol	Human podocytes stimulated by AGE (taken from the serum of diabetic patients with diabetic neuropathy)	Thymol restored the expression of RhoA, ROCK, vimentin, nephrin, and podocin and the phosphorylation of p65 and I*κ*B*α*. It inhibited the induction of proinflammatory cytokines and cell apoptosis. Thymol improves migration capacity in human podocytes induced by AGEs.	[53]
Thymol	Isolated lenses from goat eyes	Thymol stopped the progression of high-glucose-induced cataracts through its antioxidant and aldose reductase (AR) inhibitory activities	[54]

PI3K: phosphatidylinositol-3-kinase; AKT: protein kinase B; NOX4: NADPH oxidase type 4; AMPK: AMP-activated protein kinase; HepG2: human hepatocellular carcinoma cell line; AGEs: advanced glycation end products; 3T3-L1: mouse preadipocyte cell lines; PPAR*γ*: peroxisome proliferator-activated receptor *γ*; SREBP-1c: sterol regulatory element binding protein; TNF-*α*: tumor necrosis factor-alpha; IL-6: interleukin-6; MCP-1: macrophage chemotactic protein-1; ROS/EROS: reactive oxygen species; MAPK: mitogen-activated protein kinase; ERK-1: extracellular signal-regulated protein kinase; JNK: c-Jun N-terminal kinase; NRK-52E: rat proximal renal tubular cells; UCP2: uncoupling protein 2; Txnip: thioredoxin-interacting protein; INS-1: mouse pancreatic *β* cell line; TCF7L2: T cell factor 7 type 2; MC3T3-E1: osteoblastic cell line; NO: nitric oxide; PGC-1*α*: peroxisome proliferator-activated receptor gamma-1 coactivator; MMP-9: microglial matrix metalloproteinase 9; GSK-3*β*: glycogen synthase kinase-3*β*; IRS-1: insulin receptor-1 substrate; MMP: mitochondrial membrane potential; NF-*κ*B: nuclear factor-*κ*B; HIF-1*α*: hypoxia-inducible factor-1*α*; VEGF: vascular endothelial growth factor; NOX2: NADPH oxidase 2; Nrf2: factor 2 related to nuclear factor E2; ARE: antioxidant response element; PEPCK: phosphoenolpyruvate carboxykinase. RAGE: advanced glycation end product induction receptor.

**Table 4:** In vivo antidiabetic effects of monoterpenes.

Monoterpenes	Study model/dose(s)	Effects/markers	References
Beta-pinene	Alloxan-induced diabetic rats—25, 50, 100, and 200 mg·kg^−1^	Beta-pinene decreased plasma glucose, triglyceride, VLDL, LDL, and HDL levels, when compared to those of the control group. Carrageenan induced paw edema and leukocyte migration in the peritoneum.	[55]
Borneol	STZ-induced diabetic rats—25 and 50 mg·kg^−1^	Increase in the biochemical indices, i.e., fasting blood glucose concentration, glycated hemoglobin, urea, alanine aminotransferase, aspartate aminotransferase, malondialdehyde concentration, total cholesterol, triglycerides, low-density lipoprotein cholesterol, very low-density lipoprotein cholesterol, and atherogenic index, with a significant decrease in body weight, plasma insulin, HOMA-*β*-cell functioning index, glycogen, high-density lipoprotein cholesterol, and antioxidant enzyme activities, i.e., superoxide dismutase, catalase, and reduced glutathione	[56]
Carvacrol	HFD-induced C57BL/6J mice—20 mg/kg	Activation of IR, IRS, PI3K, and Akt/PKB. Induce STAT3 and SOCS. Suppress TC, TG, elevated phospholipids and FFAs, VLDL-C, and LDL-C in plasma and tissues. Suppress inflammatory cytokines in liver tissue (TNF and IL-6) and increase high-density lipoprotein cholesterol (HDL-C).	[57]
Carvacrol	HFD-induced type 2 diabetic C57BL/6J mice—20 mg/kg	The activities of carbohydrate metabolic enzymes such as glucose-6-phosphatase and fructose-1,6-bisphosphatase increased, whereas glucokinase and glucose-6-phosphate dehydrogenase activities decreased in the liver of HFD mice. Normalize liver markers (ASP, ALA, ALP, and GGT).	[58]
Carvacrol	Rats with cerulein-induced pancreatic injury—50, 100, and 200 mg/kg	Mechanism of antioxidant action	[59]
Carvacrol	STZ-induced diabetic rats—75 mg/kg	Carvacrol decreased Bax and increased Bcl-2 in gene and protein expression levels. Reduced germ cell apoptosis.	[60]
Carvacrol	STZ-induced diabetic C57BL/6J mice—10, 20, and 40 mg/kg/day	A significant improvement in glucose tolerance and a significant decrease in the plasma level of TG were observed in carvacrol-treated diabetic mice at a dose of 20 mg/kg. Reduced the plasma level of LDH but not AST, ALT, or ALP, compared with that in the vehicle-treated diabetic group. The activity of hexokinase (HK), 6-phosphofructokinase (PFK), and citrate synthetase (CS) was increased.	[61]
Carvacrol	Diabetic C57BL/KsJ-db/db male mice—10 mg/kg	Carvacrol improved blood glucose and insulin resistance of T2DM db/db mice	[62]
		The serum levels of TC, TG, and LDL-C were markedly reduced, whereas HDL-C levels were significantly increased	
		Decreased serum ALT and AST levels	
		Protective effects on the liver in T2DM db/db mice, which could be related to insulin, TLR 4/NF-*κ*B, and AKT1/mTOR signaling pathways	
Carvone	STZ-induced diabetic rats—50 mg·kg^−1^	Decline in the levels of plasma glucose, HbA1c, and significant improvement in the levels of Hb and insulin. The reversed activities of carbohydrate metabolic enzymes, enzymic antioxidants, and hepatic marker enzymes in diabetic rats were restored to near normal level by the administration of carvone.	[63]
Catalpol	Diabetic db/db mice—200 mg/kg	Lowered blood glucose and improved insulin sensitivity via activation of phosphatidylinositol-3-kinase (PI3K)/protein kinase B (AKT) pathway. Exhibited enhanced myogenesis and increased myogenic differentiation (MyoD), myogenin (MyoG), and myosin heavy chain (MHC) expressions.	[29]
Catalpol	STZ-induced diabetic rats—10 mg/kg	The treatment improved diabetes-associated impaired renal functions	[64]
		Reduced the elevated Grb10 expression in diabetic kidneys, suppressing IGF-1/IGF-1R signaling pathway	
Catalpol	db/db mice—25, 50, 100 and 200 mg/kg	Hypoglycemic and therapeutics effect via modulation of various gene expressions such as SOCS3, Irs1, Idh2, and G6pd2	[65]
Catalpol	C57BL/6J mice with type 2 diabetes induced by the combination of HFD and STZ—100 and 200 mg/kg	Regulation of the AMPK/NOX4/PI3K/AKT pathway	[30]
Catalpol	Mice fed HFD—100 mg/kg	Reduced mRNA expressions of M1 proinflammatory cytokines, but increased M2 anti-inflammatory gene expressions	[66]
		Suppressed the c-Jun NH2-terminal kinase (JNK) and nuclear factor-kappa B (NF-*κ*B) signaling pathway	
Catalpol	Diabetic mice induced by the combination of HFD and STZ—50, 100, and 200 mg/kg	Catalpol decreased fission protein 1 and protein 1 expression as well as increased mitofusin 1 expression in HepG2 cells	[31]
Catalpol	Rats with type 2 diabetes induced by the combination of HFD and STZ—10, 50, and 100 mg/kg	Negative regulation of Nox4 and p22phox expression, inhibiting the oxidative stress reaction response	[67]
Citronellol		Citronellol improved the levels of insulin, Hb, and hepatic glycogen with significant decrease in glucose and HbA1C levels	[68]
	Diabetic rats induced by STZ—25, 50, and 100 mg/kg	The altered activities of carbohydrate metabolic enzymes and hepatic and kidney markers were restored to near normal	
Cymene	STZ-induced diabetic rats—20 mg/kg	Inhibition of glycation. Improves HbA1c and nephropathic parameters (such as albumin excretion rate, serum creatinine, and creatinine clearance rate).	[33]
p-Cymene	STZ-induced diabetic rats—25, 50, and 100 mg/kg	Improve serum levels of Glu, total cholesterol (TC), triglycerides (TG), high-density lipoprotein cholesterol (HDL-c), low-density lipoprotein (LDL), very-low-density lipoprotein (VLDL), alkaline phosphatase (ALP), alanine aminotransferase (ALT), aspartate aminotransferase (AST), malondialdehyde (MDA), and the expression of mTOR, Akt, and phospho-Akt protein	[69]
Eucalyptol	Diabetic db/db mice—10 mg/kg	It increased the expression of nephrin, podocin, FAT-1, CD2AP, *α*-actinin-4, and integrin. Blocked ERK-c-Myc signaling.	[70]
Genipin	HFD-induced obese rats—12.5 and 25 mg/kg	Genipin increased the levels of PRD1-BF1-RIZ1. Inhibited the gene expressions of activin receptor kinase 7, TNF-*α*, and IL-6.	[71]
Genipin	Alloxan-induced diabetic rats—25, 50 and 100 mg/kg	Genipin reduced disturbance in glucose metabolism, ACT, lipid metabolism, and amino acid metabolism	[72]
Genipin	Diabetic rats—25, 50, and 100 mg/kg	Regulation of methylamine metabolism, energy metabolism, and amino acid metabolism Nephroprotective effect	[73]
Geniposide	STZ-induced diabetic rats—25, 50 and 100 mg/kg	Inhibition of ICAM-1, TNF-*α*, IL-1, and IL-6 expression and inhibition of NF-kB activation by NF-*κ*B, IKK*α*, and I*κ*B*α* expression	[74]
Geniposide	Diabetic C57BL/KsJ-db/db mice—20 and 40 mg/kg	Inhibition of Rho, ROCK1, ROCK2, p-NF-*κ*Bp65, and p-I*κ*B*α* expression	[75]
Geniposide	HFD-induced diabetic mice—10 and 100 mg/kg	Inhibited transcription of G6PC and PEPCK. Suppress hepatic gluconeogenesis by regulating the AKT-FOXO1 pathway	[76]
Geniposide	STZ-induced diabetic C57BL/6 mice)—50 mg/kg	Geniposide increased the activities of PKA and GSK-3*β*, possibly modulating AMPK and AKT pathways, efficiently improving renal dysfunction, and ameliorating the progression of DN	[77]
Geniposide	STZ-induced diabetic C57BL/6 mice—50 mg/kg/day	GEN inhibited ROS accumulation, NF-*κ*B activation, Müller cell activation, and inflammatory	[78]
Geniposide	STZ-induced diabetic rats—200, 400, and 500 mg/kg	Geniposide significantly reduced inflammatory cell infiltration and proliferation of fibroblasts in the central lesion regions, and the levels of proinflammatory factors (tumor necrosis factor-a (TNF-a), interleukin-1b (IL-1b)) and IL-6 were significantly reduced	[79]
Geraniol	STZ-induced diabetic rats—100, 200, and 400 mg/kg	Administration for 45 days significantly improved the levels of insulin and Hb and decreased plasma glucose and HbA1C	[80]
Geraniol	STZ-induced diabetic rats—150 mg/kg	Geraniol suppressed the exaggerated oxidative stress as evidenced by preventing the increase in 8-isoprotane. Geraniol partially reduced hyperglycemia and prevented the hypercholesterolemia but did not affect the serum level of adiponectin in diabetic animals Potent protective effect against cardiac dysfunction	[81]
Geraniol	STZ-induced diabetic rats—100 mg/kg	Reduced oxidative markers, decreased levels of PC, Ca2+, and AChE, restored enzyme activities, SDH, and CS	[82]
Gentiopicroside	HFD-induced diabetic mice—10 and 100 mg/kg	Regulation of the AKT-FOXO1 pathway	[76]
D-Limonene	STZ-induced diabetic rats—50 mg/kg	The blood glucose levels were found significantly lower at the 21st and 28th days of treatment The serum AST and GGT levels, LDL, total cholesterol, and triglyceride levels in the D-limonene treated diabetic group were found to be significantly lower. The CAT, SOD, GSH-Px enzyme activities, and GSH levels in plasma, liver, and kidney increased	[83]
Loganin	STZ-induced diabetic mice—20 mg/kg	Reduces AGE levels and negatively regulates mRNA and protein expression of receptors for AGEs. Inhibition of AGE pathway.	[84]
Loganin	STZ-induced diabetic rats—40 mg/kg	Inhibition of proinflammatory cytokines	[85]
Loganin	STZ-nicotinamide-induced diabetic Sprague Dawley rats—5 mg/kg	Loganin improved PDN rats' associated pain behaviors (allodynia and hyperalgesia), insulin resistance index (HOMA-IR), and serum levels of superoxide dismutase (SOD), catalase, and glutathione. Loganin also reduced pain-associated channel protein CaV3.2 and calcitonin gene-related peptide (CGRP) in the surficial spinal dorsal horn of PDN rats. Loganin inhibited oxidative stress and NF-B activation and decreased the levels of mRNA and protein of proinflammatory factors IL-1 and TNF-*α*	[86]
Menthol	STZ-nicotinamide-induced diabetic rats—25, 50, and 100 mg/kg	Suppression of pancreatic *β* cell apoptosis, increased Bcl-2 expression, and reduced Bax expression. Modulation of glucose-metabolizing enzymes.	[87]
Myrtenal	STZ-induced diabetic rats—80 mg/kg	Myrtenal decreases plasma glucose, and increases plasma insulin, as well as upregulates IRS2, Akt, and GLUT2 protein expressions in liver, and IRS2, Akt and GLUT4 protein expressions in skeletal muscle	[88]
Myrtenal	STZ-induced diabetic rats—20, 40, and 80 mg/kg	The altered activities of the key metabolic enzymes involved in carbohydrate metabolism such as hexokinase, glucose-6-phosphatase, fructose-1,6- bisphosphatase, glucose-6-phosphate dehydrogenase, and hepatic enzymes AST, ALT, and ALP levels of diabetic rats were significantly improved by the administration of myrtenal in STZ-induced diabetic rats	[89]
Myrtenal	STZ-induced diabetic rats—80 mg/kg	Myrtenal improved plasma glucose levels while lowering levels of lecithin cholesterol acyltransferase (54.61 moles of cholesterol esterified/h/L), high-density lipoprotein (29.12 mg/dL), and pancreatic insulin (97.48 ng/mg)	[90]
Paeoniflorin	Mouse—70 and 140 mg/kg	Cardioprotection possibly by TRPV1/CaMK/CREB/CGRP signaling pathway	[91]
Paeoniflorin	STZ-induced diabetic mice—20 and 40 mg/kg	Induced suppression of cytokine signaling 3 (SOCS3) expression and reduced MMP-9 activation in BV2 cells	[44]
Paeoniflorin	Diabetic rats induced by a diet rich in sucrose, fat and low dose of streptozotocin—15 and 30 mg/kg	Paeoniflorin was correlated with its abilities of reducing the brain inflammatory cytokines (IL-1*β* and TNF-*α*), decreasing suppressor of cytokine signaling 2 (SOCS2) expressions, and promoting insulin receptor substrate-1 (IRS-1) activity. Additionally, we also found that paeoniflorin administration significantly promoted the phosphorylation levels of protein kinase B (Akt) and glycogen synthase kinase-3*β* (GSK-3*β*).	[92]
Paeoniflorin	C57BL/6J mice with STZ-induced diabetic nephropathy—25, 50, and 100 mg/kg	Inhibition of toll-like receptors (TLR-2) pathway	[93]
Paeoniflorin	db/db mice with type 2 diabetic nephropathy—15, 30, and 60 mg/kg	Inhibit macrophage infiltration and activation by blocking the TLR2/4 signaling pathway	[94]
Paeoniflorin	Sprague Dawley rats with fructose-induced hepatic steatosis—10, 20, and 40 mg/kg	Activation of LKB1/AMPK and in insulin signaling, activation of *β*-oxidation and glycogenolysis	[95]
Paeoniflorin	Mice with cognitive deficits induced by intracerebroventricular injection of STZ in mice—10 mg/kg	Positive regulation of PI3K and Akt protein expression, while negatively regulating IRS-1 protein expression	[96]
Paeoniflorin	Type 1 diabetic mice induced by STZ—25, 50, and 100 mg/kg	Inhibition of the JAK2/STAT3 signaling pathway	[97]
Paeoniflorin	STZ-induced diabetic rats—10 *μ*M	PF enhanced peroxiredoxin 3 (Prx3), mitochondrial processing peptidase *α* (PMPCA) expression, small ubiquitin-related modifier 1 (Sumo1) to enhance mitochondrial protein processing of Trx2, and mitochondrial protein processing of Trx2. In the sciatic nerve of DPN rats, PF elevated the levels of Trx2, TrxR2, and Prx3. PF increased the Trx2, TrxR2, and Prx3 levels in sciatic nerve of DPN rats.	[98]
Swertiamarin	STZ-induced diabetic rats—15, 25, and 50 mg/kg	Swertiamarin decreased fasting blood glucose, HbA1c, TC, TG, and LDL and increased the levels of hemoglobin, plasma insulin, TP, body weight, and HDL significantly	[99]
Thymol	C57BL/6J mice with HFD-induced DT2—10, 20, and 40 mg/kg	Thymol increased activities of LCAT and LPL and decreased activities of HMG-CoA reductase	[100]
Thymol	High-fat diet (HFD)-induced diabetic C57BL/6J mice—40 mg/kg	Thymol inhibited the activation of transforming growth factor *β*1 (TGF-*β*1) and vascular endothelial growth factor (VEGF)	[101]
Thymol	C57BL/6J mice on HFD diet—20 and 40 mg/kg	Thymol downregulated the level of P-Ser307 IRS-1, hence enhancing the expression of P-Ser473 AKT and P-Ser9 GSK-3*β*	[102]
Thymol	STZ-induced diabetic rats—10 and 20 mg/kg	Antidiabetic and neuroprotective Biomarkers such as SOD, NO, LPO, Na + K + ATPase, and TNF-*α* further confirmed the protective action of thymol in diabetic neuropathy	[103]

STZ: streptozotocin; HFD: high-fat diet; TC: total cholesterol; TG: triglycerides; VLDL: very low-density lipoprotein; LDL: low-density lipoprotein; HDL: high-density lipoprotein; HbA1c: glycated hemoglobin; ALT: alanine aminotransferase; AST: aspartate aminotransferase; FA: alkaline phosphatase; SOD: superoxide dismutase; CAT: catalase; GSH: glutathione; MDA: malondialdehyde; PI3K: phosphatidylinositol-3-kinase; AKT/PKB: protein kinase B; Grb10: protein linked to the growth factor receptor 10; IR: insulin receptor; IRS: insulin receptor substrate; IRS-1: insulin receptor-1 substrate; IRS2: insulin receptor-2 substrate; STAT3: signal transducer and transcription activator 3; SOCS: cytokine signaling suppressor expression; SOCS3: cytokine signaling suppressor 3 expression; TNF: tumor necrosis factor; IL-6: interleukin 6; IL-1: interleukin 1; LDH: lactate dehydrogenase; HK: hexokinase activity; PFK: 6-phosphofructokinase; CS: citrate synthase; NF-*κ*B: nuclear factor-*κ*B; TD2: type 2 diabetes; TLR 4: toll-like receiver 4; IGF-1: insulin-like growth factor 1; IGF-1R: insulin-like growth factor receptor 1; IDH2: isocitrate dehydrogenase 2 (NADP+); G6PD2: glucose-6-phosphate dehydrogenase 2; HepG2: human hepatocellular carcinoma cell line; TNF-*α*: tumor necrosis factor alpha; ICAM-1: intercellular adhesion molecule-1; JNK: c-Jun N-terminal kinase; PEPCK: phosphoenolpyruvate carboxykinase; GSK3: glycogen synthase kinase-3; Nrf2: factor 2 related to the nuclear factor; PC: protein carbonyls; SDH: succinate dehydrogenase; AChE: acetylcholinesterase; MMP-9: microglial matrix metalloproteinase 9; FFAs: free fatty acids; LCAT: lecithin cholesterol acyltransferase; LPL: lipoprotein lipase; SREBP-1c: sterol regulatory element binding protein; VEGF: vascular endothelial growth factor; TGF-*β*1: transforming growth factor *β*1; NO: nitric oxide; LPO: neutral lipid peroxidase.

**Table 5:** Patents related to the use of monoterpenes in diabetes therapy.

Title	IPC	Year	Country	Patent purpose	Reference
Compositions of monoterpenoids for stimulating the immune system	A61K31/045	2012	France	Pharmaceutical composition containing monoterpenoids, intended for the treatment of immune disorders, associated with conditions such as diabetes, alcohol or drug abuse, or obesity	[111]
Extract of *Toona sinensis* from supercritical fluid extraction for treating diabetes and metabolic diseases, the preparation method and the use thereof	A61K36/58	2011	China	*Toona sinensis* extract should be used as a food supplement or in pharmaceutical compositions. It can lower blood sugar levels, promote lipid breakdown, and treat metabolic disorders.	[112]
Synergistic composition of (−)-hydroxycitric acid with monoterpene and a method to enhance satiety	A61K31/194	2011	EUA	Pharmaceutical or synergistic food composition comprising (−)-hydroxycitric acid, its salts, amides, and esters together with monoterpenes to increase satiety and help control obesity and prevent diabetes	[113]
Use of monoterpenes compound in preparation of medicine against diabetes mellitus	A61K31/015	2006	China	An application of the monoterpene compounds, especially d-limonene, in the preparation of the drug in oily liquid form for the treatment of diabetes is disclosed	[114]
Nutritional and therapeutical preparations having antioxidant activity	A23D7/00	2004	EUA	Pharmaceutical and dietary compositions for the treatment and prevention of aging processes and related conditions, among them diabetes, comprising a lipid blend rich in polyunsaturated fatty acids and vitamins, in combination or not with monoterpenes and/or sesquiterpenes	[115]

IPC: International Patent Classification.

## Data Availability

The data used to support the findings of this study are available from the corresponding author upon request.
